# Non-invasive assessment of equine muscular function: A case study

**Published:** 2013-07-12

**Authors:** K.H. Riis, A.P. Harrison, K. Riis-Olesen

**Affiliations:** 1 *IKVH, Faculty of Health & Medical Sciences, Copenhagen University, Grønnegaardsvej 7, 1870 Frederiksberg C, Denmark*; 2 *Hillerød Hestedyrlæger, Baunevej 17, Bendstrup, 3400 Hillerød, Denmark*

**Keywords:** Accelerometry, Acoustic myography, Bioimpedance analysis, Lameness

## Abstract

Assessment of muscle function after an injury or during recovery is of great importance in the veterinary field. Accelerometry, bioimpedance analysis and mechanomyography/acoustic myography have been used to assess human muscular problems, but have not been applied to the veterinary clinic. We report the clinical use of these techniques in a 12-year-old Danish Warmblood horse presenting with recurring and shifting lameness. Acoustic myography, assessing both the amplitude and frequency of active muscles, was employed to locate the specific area of muscle injury, the right hip, which exhibited minimal fibre recruitment giving rise to considerable weakness. This specific region was assessed by accelerometry which revealed a normal step interval for the injured leg when compared with the contralateral, but a weaker acceleration and strike force. Finally, an assessment of muscle resistance (R) and reactance (Xc) using bioimpedance confirmed a regional loss of muscle mass and a loss of cellular integrity compared with the contralateral limb.

## Introduction

Riding of horses, whether it be professional or amateur horse racing, dressage, steeplechase or endurance for example, often leads to a number of muscular problems ranging from exercise related muscular stiffness to more complex problems involving some level of injury (Walmsley *et al.*, 2010), that may first become apparent when lameness occurs. Muscle injuries are typically classified according to their severity and are classified as one of three grades: Grade I, mild: strain/contusion representing a tear of only a few muscle fibres with minor swelling and discomfort accompanied by no or minimal loss of strength or restriction of movement, grade II, moderate: strain/contusion with a greater degree of damage of the muscle with a clear loss of function (ability to contract), and grade III, severe: a tear extending across the entire cross section of the muscle and, thus, resulting in a complete loss of muscle function (Järvinen *et al.*, 2005).

Sound/acoustic recording from skeletal muscles in humans has been shown to be a useful method for a few decades (Stokes and Blythe, 2001). Indeed, the use of Piezoelectric crystals enabling human muscle recordings transdermally has resulted in a number of advances in the field of acoustic myography (AMG), often referred to as mechanomyography (MMG) or accelerometer myography (Madeleine *et al.*, 2001; Herda *et al.*, 2010; Alves and Chau, 2011; Qi *et al.*, 2011). Yet, it is only within the last year and a half that microphones and contact transducers as well as recording systems have become available in required sizes, quality, weight and durability so as to enable them to be used as a clinical tool for the assessment of musculoskeletal complaints.

Bioimpedance analysis (BIA) uses the components of impedance (Z), resistance (R) which is the opposition to the flow of an alternating current through intra- and extracellular ionic solutions, and reactance (Xc) which is the delay in the passage of current through the cell membranes and tissue interfaces (Van Der Aa Kuhle *et al.*, 2006). Resistance is inversely related to the fluid content and Xc indicates cell membrane mass, function and interface. In this way, BIA enables characterization or classification of relative changes in hydration and cell health/damage in a non-invasive fashion (Nescolarde *et al.*, 2013).

This study therefore aims to evaluate the suitability and diagnostic potential of three non-invasive techniques, namely AMG, accelerometry and BIA used in conjunction as a tool to assess a case of recurring and shifting lameness in a horse.

## Case Details

Considerable care was taken to ensure that the horse was not subjected to any form of stress by our non-invasive measurements. Furthermore, the owners were informed of our measurements and a prior consent was obtained before we began our assessment.

### Subject

The horse, a 12-year-old Danish Warmblood, was measured at the level of S1-S2 and was found to be 169.5 cm in height, and had a calculated body weight of 560 kg. After a pre-purchase examination the horse was bought by the current owners, despite the fact that the hip musculature appeared to be asymmetrical (right side less muscle mass *cf* the left side). Three months after being purchased, a period of prolonged lameness ensued. Initially, the horse showed symptoms of fetlock (metacarpo/metatarsophalangeal joint) arthritis in the left fore limb (thoracic limb). A few months later, bone spavin in the right hind limb (pelvic limb) which is a bony growth within the hock joint as the result of osteoarthritis was diagnosed and facet-joint problems in the back were also noted. Subsequently, the horse became lame in the left hind limb due to suspensory ligament desmitis (SLD). Then a few months later, another case of SLD was reported, this time in the right front limb. The horse then continued to be lame in the left front and right hind limbs for the next 6 months.

In total, a prolonged period of lameness lasting for two and a half years passed by from the date of purchase until the present muscle measurements. It should be noted that this horse was examined by our veterinary clinic as per the wishes of the owners who wanted a second opinion.

### AMG

A custom built AMG unit (MyoDynamik ApS – *www.myodynamik.com*), capable of recording at a distance of 100 m and with a sampling rate of 96000 Hz, was placed on the horse at the point of the withers and mounted as part of a customized training girth. The unit was set to record muscle sounds transdermally from the *m. Gluteus medius* of the hip.

The recorded data were analyzed for their frequency and amplitude parameters because the central nervous system (CNS) coordinates muscle function by increasing or decreasing the recruitment and synchronization of motor units (equates to signal amplitude), as well by altering the frequency with which active motor units fire (equates to signal frequency: Salomons *et al.*, 2012).

### Accelerometry

An accelerometer (Waveland, US), was attached to the horse using animal polster-plast (Snøgg, N-4671 Kristiansand, N) just above the vertebral column at the level of the hips. The unit was programmed to record at 100 Hz (100 samples / second) and it was sensitive to directional acceleration above 0.1 g and up to a maximum of 8.0 g.

The recorded data were analyzed for their acceleration with a particular emphasis on the “g” forces produced by the hips during trotting e.g. the rise and fall of the left and right hips in relation to the vertebral column. These data reveal critical information as to the coordination of the hind limbs and the acceleration of the left and right-hand sides of the horse during movement.

### BIA

The horse was restrained in a standing position and kept free of all metal surfaces. A precisely determined anatomical area, which had been previously tested on a number of race horses and other well trained horses (n=10) to provide control data, was then prepared by shaving and a conductive paste was applied (Ten20, Aurora US) after which four custom made pure platinum electrodes (1 × 3 cm) were placed on the prepared sites. The region covering *m. Gluteus medius* at the level of S2-S3 through to the mid *m. Biceps femoris* was assessed in this manner.

A BIA unit (ImpediVET BIS 1, Pinkenba, AU) providing 800 μA of current was subsequently attached to the electrodes and recordings of the resistance (R) and reactance (Xc) at 256 frequencies ranging from 3 kHz to 850 kHz were made. The BIA unit was set to record six consecutive measurements and the mean of these values was used. Using this approach, any slight movement artifacts or changes in the resistance and reactance values due to cable movement, change in the stance, body or electrode movement were minimized.

## Discussion

The horse presented to our clinic had previously been diagnosed with a number of complaints ranging from arthritis of the fetlock joint of the left fore limb to SLD and sesamoiditis, as well as lameness. We believe that these diagnoses were based on insufficient information regarding the precise cause and origin of the horses primary injury, and that the symptoms were instead indicative of “referred” or “secondary” problems. Such “referred” problems might for example arise from a compensatory shift in balance, or a change in use of muscle groups associated with the hind limbs and vertebral column, resulting in muscle strain and joint pain in other regions of the body.

The data from the AMG sensors placed on the hip muscle (*m. Gluteus medius*) revealed a clear difference in terms of amplitude between the left and right hind limbs. The left hind limb presented with a higher signal that exhibited an amplitude range of 10.5 to 12.0 mV compared with that of the right hind limb (0.5 mV). Interestingly the frequency for the AMG signal in this muscle was not appreciably different between the two hind limbs (Table 1).

**Table 1:** Measured AMG values for the left-hand and right-hand sides of the horse during a period of free trot. The AMG units were placed above the *m. Gluteus medius* of the hip (see methods for details) and recordings were made at a sampling rate of 96000 Hz. Values are presented as the Mean ± SD.

Repeat Measurements	Left-hand side		Right-hand side	
	*F. (Hz)	*A. (mV)	*F. (Hz)	*A. (mV)
Trot – Free No.1	106.6	12.0	141.7	0.5
Trot – Free No.2	117.9	11.8	152.6	0.5
Trot – Free No.3	115.0	11.5	158.3	0.5
Trot – Free No.4	111.0	10.5	140.5	0.5
Mean Value	112.6±4.9	11.5±0.6	148.3±8.6	0.5±0.0

* F. = Frequency, A. = Amplitude

**Fig. 1:**
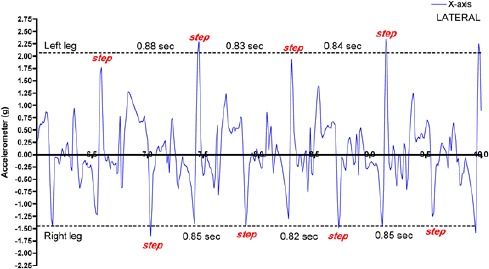
The accelerometer placed on the vertebral column at the level of the hips revealed not only the step intervals (values between the red “step” text e.g. 0.88 sec) indicating the time between each foot-fall for the hind limbs, but also indicated the relative acceleration of the two hind limbs at the level of the spinal column. The mean acceleration value for the individual “steps” in the upper part of the figure (Left Hind limb) as denoted by the dotted horizontal line was 2.07 g, whilst for the lower part of the figure (Right Hind limb) as denoted by the dotted horizontal line was 1.47 g. Note that despite both hind limbs having a similar step interval (0.85 sec Left & 0.84 sec Right), the acceleration (g-force) for the right hind limb was approximately 30% lower than that of the left hind limb.

The data recorded by the accelerometer (X-axis – lateral) revealed typical signal values for this recording site (vertebral column - hips). The step interval, which denotes the time between foot-falls, was found to be very similar (~ 0.85 sec) for both the left hind limb (upper part of Fig. 1) and the right hind limb (lower part of Fig. 1).

However, it was interesting to note that the right hind limb consistently delivered less acceleration (~ 1.25 to 1.7 g) than the left (~ 1.8 to 2.35 g). Indeed this weaker form of movement represented ~ a 30% lower acceleration compared to that of the left hind limb.

**Fig. 2:**
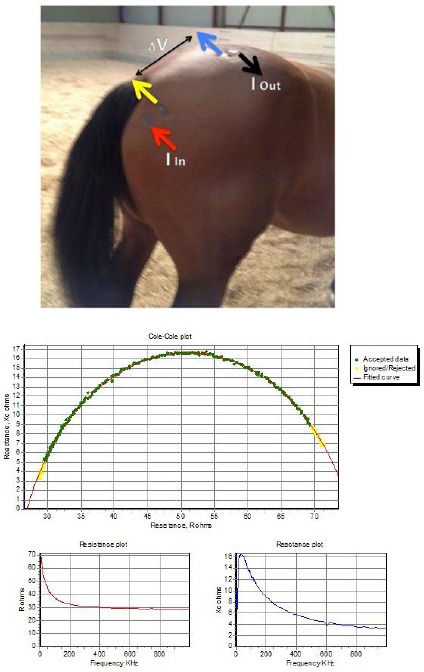
BIA current (I: red and black arrows) and voltage (V: yellow and blue arrows) electrodes were placed as shown (upper panel) on both the left-hand and right-hand sides of the horse. The electrode sites were first shaved and thereafter a conductive paste was applied before application of the electrodes themselves. Alternating current (800 μA) was applied across the red and black electrodes (entry point “I In” and exit point “I Out”). The fall in voltage across this region was measured as the delta between the yellow and blue electrodes (ΔV). The measurements revealed a typical Cole-Cole plot and normal Resistance (R) and Reactance (Xc) values (lower panel).

Likewise, the data recorded by the BIA unit revealed typical signal values (Fig. 2).

The signals exhibited a clear Cole-Cole plot, a widely used semi-circular graphical representation of frequency-dependent complex dielectric functions such as impedance, reactance and resistance for the 256 frequencies used, enabling the collection of normal resistance and reactance data for this region of the horse.

Using this technique, a clear difference in both the R and Xc between the left and right hind limbs was revealed (Table 2).

**Table 2:** Measured BIA values for the left-hand and right-hand sides of the horse, measured between the *m. Gluteus* medius and the mid *m. Biceps femoris* (see methods for details). Values are shown for the 50 kHz frequency measurement. The horse, which was 12 years old, was measured as having a height, at the level of S1-S2, of 169.5 cm and a calculated body weight of 560 kg using a weight-tape.

	Left-hand side	Right-hand side
Resistance (R) Ω	55	45
Reactance (Xc) Ω	20.0	16.5

The AMG data indicate that the right hind limb muscle (*m. Gluteus medius*) has a very low amplitude compared to the left hind limb, a difference that despite some degree of compensation by an increase in the rate of contraction (~ 150 Hz compared to 110 Hz) would almost certainly result in a much weaker contraction in this muscle, which is used to flex the hip joint and protract and abduct the hind limb (Salomons *et al.*, 2012).

Indeed, it is known that the amplitude of such signals is highly dependent on the number of muscle fibres recruited, their size and their degree of coordination (Salomons *et al.*, 2012).

This imbalance in muscular strength at the region of *m. Gluteus medius* between the right and left hind limbs, as assessed by AMG, did not appear to affect the step interval.

We found that the accelerometer data showed a perfect symmetry of ~ 0.85 sec per step during trotting for both the right and left hind limbs. These values compare well with the published data (Walker *et al.*, 2012).

Yet, in support of the AMG differences, the accelerometer data revealed an imbalance in terms of the rate of limb acceleration, which will also affect the strike force when the limbs contact the ground, a difference that one might view as being contributory to the observed symptoms of lameness.

Finally, the BIA data were assessed for this same anatomical region. The finding of a combined 20% reduction in the R and Xc values on the right hind limb when compared to the left hind limb at the region of the *m. Gluteus medius* to mid *m. Biceps femoris* would indicate not only a loss of muscle mass, but also a loss of cellular integrity with an impaired ability to maintain cell membrane mass as well as membrane function – making our values comparable to those typically seen with a muscle injury (grade II) (Järvinen *et al.*, 2005; Nescolarde *et al.*, 2013).

## Conclusion

We have examined a horse that was diagnosed with arthritis of the fetlock joint of the left fore limb, SLD and sesamoiditis, as well as lameness in the right hind limb. We were able to show, using AMG, that the right hind limb of this particular horse had a very low signal amplitude, despite a normal firing frequency, and that this could most likely be attributed to muscle fibre loss/atrophy.

The accelerometer data confirmed that despite a balanced step interval, the right hind limb had a 30% lower rate of acceleration compared to the left hind limb, and the BIA indicated that this particular region of the right hind limb had not only a lower muscle mass but also compromised muscle fibres compared with the same region of the contralateral limb.

We explicitly show that such non-invasive measurements of muscle function using a combination of AMG, accelerometry and BIA can provide a useful set of diagnostic tools for rapidly determining muscular injury. Diagnosis by the use of these methods can enhance the chances of subsequent recovery during rehabilitation of such an affected horse.
